# Effectiveness of decontamination by litter removal in Japanese forest ecosystems affected by the Fukushima nuclear accident

**DOI:** 10.1038/s41598-020-63520-8

**Published:** 2020-04-20

**Authors:** Jun Koarashi, Mariko Atarashi-Andoh, Syusaku Nishimura, Kotomi Muto

**Affiliations:** Nuclear Science and Engineering Center, Japan Atomic Energy Agency, Ibaraki, 319-1195 Japan

**Keywords:** Environmental monitoring, Environmental chemistry, Environmental impact

## Abstract

The Fukushima Daiichi nuclear power plant accident caused serious radiocesium (^137^Cs) contamination of forest ecosystems over a wide area. The removal of the forest floor litter layer has been considered a potential method for forest decontamination; however, its effectiveness remains largely unknown. We conducted a pilot-scale decontamination study in a deciduous broadleaved forest in Fukushima. The entire forest was decontaminated by removing the litter layer in July 2014, approximately 3.3 years after the accident, with the exception of two untreated plots. For three years after decontamination, we quantified ^137^Cs contamination levels in the litter and topsoil layers and in the tree leaves, in the untreated and decontaminated areas. The decreased inventories of litter materials and the litter-associated ^137^Cs in the decontaminated areas were observed only in the first year after decontamination. Generally, no decontamination effects were observed on the ^137^Cs transfer in tree leaves. The primary reason for this was the rapid shift in the main reservoir of ^137^Cs from litter layers to the underlying mineral soil, which differs from the observations in post-Chernobyl studies of European forest ecosystems. The results suggest that litter-removal decontamination can only be successful if it is implemented more quickly (within 1–2 years after the accident) for Japanese forest ecosystems.

## Introduction

The accident at the Fukushima Daiichi nuclear power plant (NPP) in March 2011 released large quantities of radionuclides into the atmosphere^1^ and consequently caused serious radioactive contamination of terrestrial ecosystems over a wide area of eastern Japan^2^. Among the radionuclides deposited onto the terrestrial ecosystems, radiocesium (^137^Cs), with a physical half-life of 30.1 years, is the primary cause for concern because ^137^Cs-contaminated ecosystems can increase the radiation exposure to the local population for many years, via both an elevated ambient dose rate in the air (external exposure) and the consumption of contaminated food products (internal exposure)^3^.

Decontamination is an important post-accident countermeasure to reduce the radiological impact of a nuclear accident on humans and ecosystems^3,4^. Although since the Fukushima NPP accident, decontamination efforts have been progressing in residential and agricultural areas, forested areas have received a low priority in the decontamination decision-making process, as in the case of the Chernobyl NPP accident^3^, and has not been carried out on a large scale^5^, even though forests provide a wide range of social, cultural, and economic values. This is because the forested areas are vast, occupying approximately 70% of the land in the heavily contaminated area^6^, but are generally not where people live permanently or regularly spend much time. Another reason may be that the forested areas in Japan are concentrated in mountainous and hilly regions with steep terrain, which makes them very sensitive areas to soil erosion. Technical, radiological, and economic difficulties associated with forest decontamination, as well as a lack of detailed understanding of the behavior of ^137^Cs in contaminated forests, are also significant factors in retarding the progress of forest decontamination^7–10^. Consequently, forest decontamination has only been applied to limited areas in Fukushima (e.g., the edges of forests) for the purpose of reducing the level of external exposure to the population in adjacent living environments^5^.

Studies conducted following the accident at the Chernobyl NPP have shown that self-decontamination of forests by natural ecosystem processes proceeds extremely slowly, with the normal net export of ^137^Cs from forest ecosystems being less than 1% per year^11–14^, indicating that forest ecosystems act as an effective long-term reservoir of deposited ^137^Cs. Despite the fact that the absolute natural losses of ^137^Cs from forest ecosystems are small, it is well documented that recycling of ^137^Cs within a forest ecosystem occurs continuously, and forest floor litter layers with less ^137^Cs-fixation potential compared with the underlying mineral soil are the key ecosystem component for the persistence of this recycling^15,16^. The recycling of ^137^Cs can lead to enduring ^137^Cs contamination in forest products and animals providing human food^17,18^. Given these findings, it is still worthwhile to consider conducting artificial interventions to the contaminated Fukushima forest ecosystems.

Potential (technology-based) decontamination methods for forest ecosystems include the removal of litter and surface soil, clear-cutting and plowing, and the application of fertilizers and other chemicals^3,19^. Among these, the removal of the litter layer is considered the primary decontamination method because it is less expensive and relatively easy to implement, with less ecological perturbation compared with other methods. However, case studies in which litter-removal decontamination has been applied in practice to European forest ecosystems after the Chernobyl NPP accident are limited^3^. Notwithstanding reports of successful reduction of the local external radiation level in European forest ecosystems by litter removal^4,20,21^, its effects on ^137^Cs recycling in forest ecosystems remain poorly understood^3^. It is reasonable to assume that the ^137^Cs behavior, and thus the decontamination efficiency, depends on the nature of forest ecosystems, which varies widely according to climate (temperature and precipitation), tree species composition, soil properties, and topography^7,15,22–25^. Because these climatological and ecological factors differ greatly between European and Japanese forest ecosystems, the findings from the post-Chernobyl studies for European forest ecosystems may not be entirely applicable in the case of the Fukushima NPP accident. Therefore, more investigation is needed to evaluate the potential effectiveness of litter-removal decontamination for forest ecosystems, particularly under the specific climatological and ecological conditions in Japan^10,26–28^.

**Figure 1:**
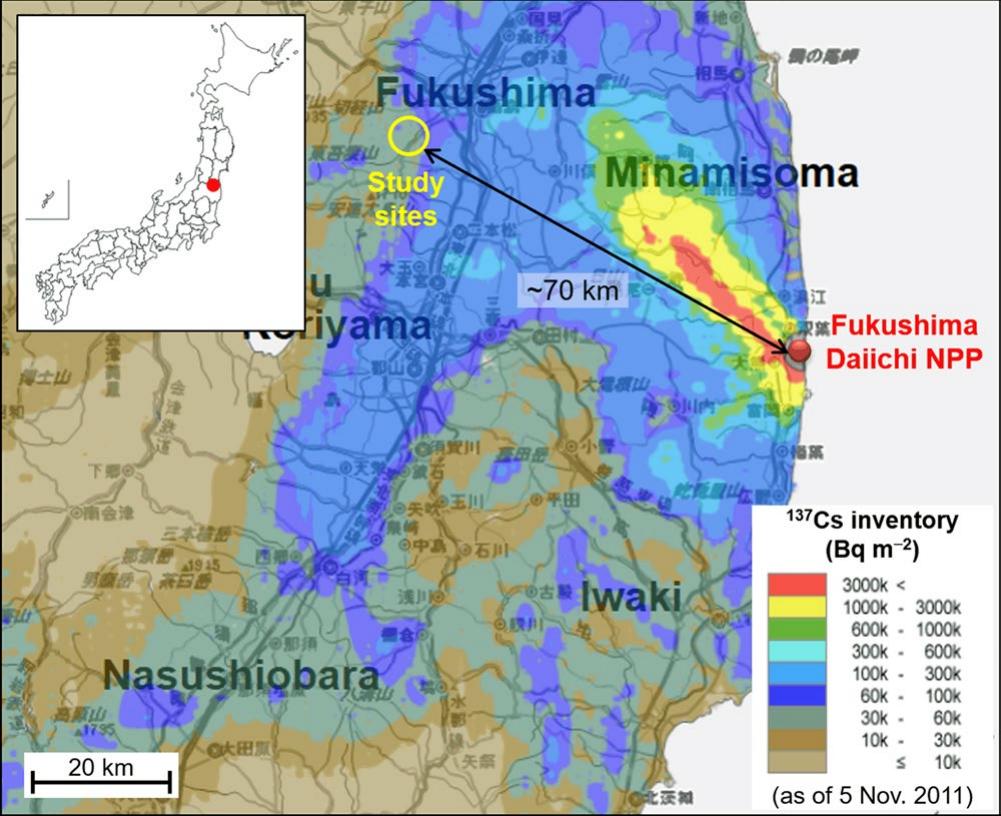
Location of the study sites. The ^137^Cs inventory map was generated using the website “Extension Site of Distribution Map of Radiation Dose, etc.,/GIS Maps” prepared by the Ministry of Education, Culture, Sports, Science, and Technology, Japan^64^.

**Figure 2:**
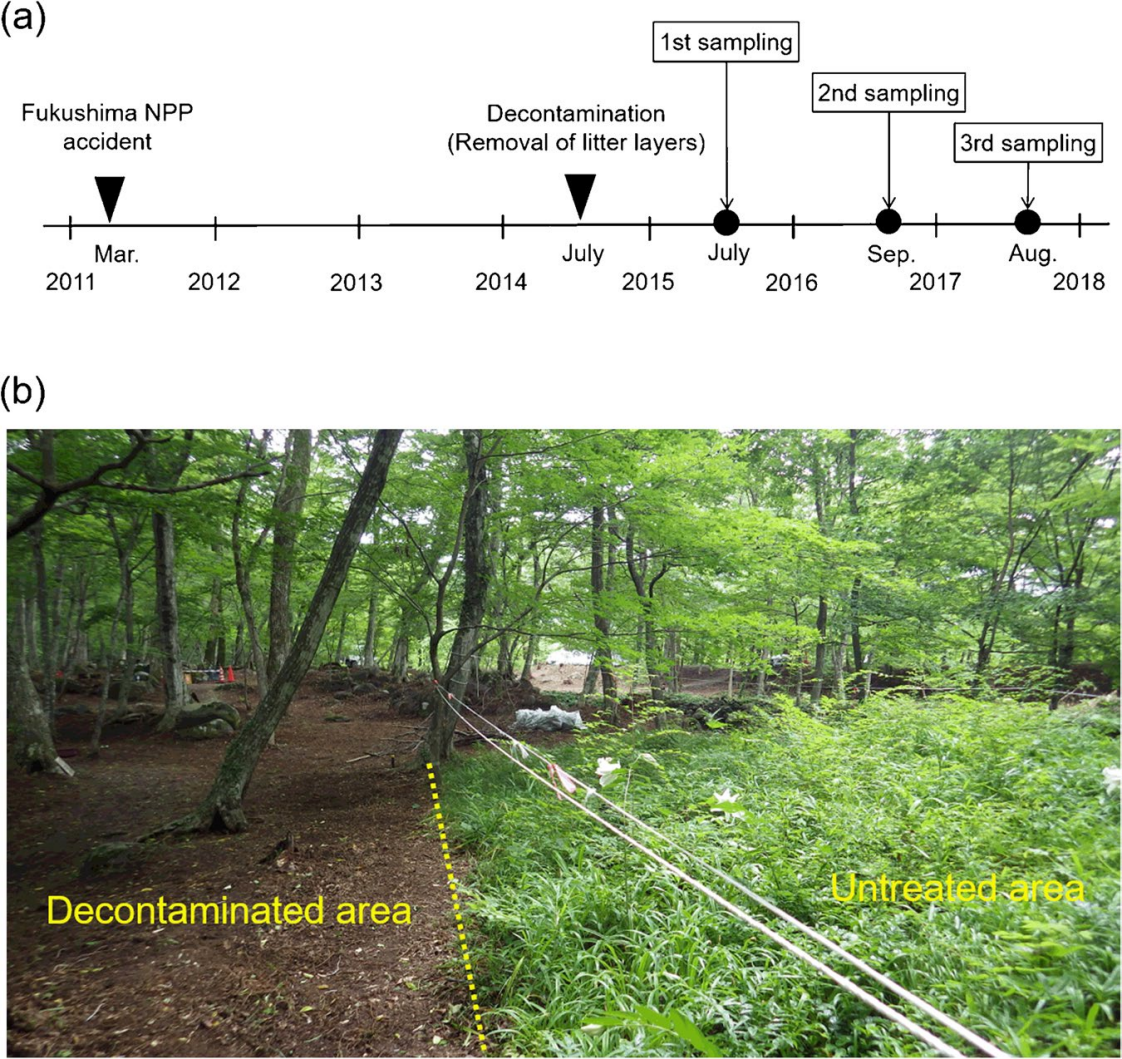
(**a**) Time sequence of decontamination operation and sample collections, and (**b**) photograph taken at the FR-1 site just after litter-removal decontamination by J. Koarashi.

Here we show results from a pilot-scale decontamination study conducted at two sites (FR-1 and FR-2) in a Japanese deciduous broadleaved forest affected by the Fukushima NPP accident (Fig. 1). At each of the sites, a plot was established as an untreated area (i.e., not decontaminated). In July 2014, the entire area of the forest was decontaminated by removing the forest floor litter and understory, with the exception of the two untreated plots (Fig. 2). In the untreated and adjacent decontaminated areas of the two sites, samples (litter, topsoil (0–5 cm), and tree leaves) were collected on three occasions over the three years following the decontamination: July 2015, September 2016, and August 2017. The collected samples were then analyzed to quantify ^137^Cs inventories in the litter and topsoil layers, ^137^Cs activity concentrations in tree leaves, and aggregated transfer factors (*T*_*ag*_) for ^137^Cs in tree leaves. Based on the results, we evaluated the effects of litter-removal decontamination on the reduction of ^137^Cs contamination and recycling in Japanese forest ecosystems.

## Results

### Litter inventory

**Figure 3:**
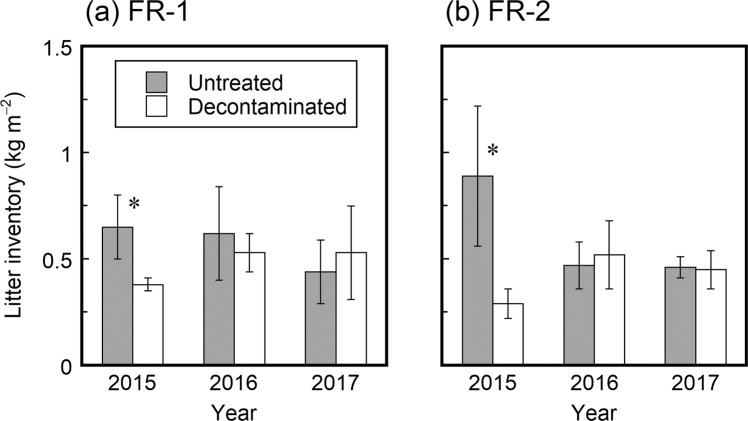
Temporal changes after decontamination (July 2014) in the inventory of litter materials in the litter layers in the untreated and decontaminated areas at (**a**) FR-1 and (**b**) FR-2 sites. Asterisks indicate statistically significant differences.

At both the FR-1 and FR-2 sites, the inventory of litter materials in the forest floor litter layer (excluding coarse woody debris) was observed to be higher in the untreated area than in the decontaminated area on the first sampling occasion (July 2015), one year after decontamination (Fig. 3). This was most likely the result of the removal of litter from the decontamination area in July 2014. However, the difference in the litter inventory between the two treatment areas was not found to be significant on the second and third sampling occasions (approximately two and three years after decontamination, respectively). The litter inventory ranged from 0.29 to 0.89 kg m^−2^ and was similar between the two sites (FR-1 and FR-2).

### Concentration of ^137^Cs in litter and topsoil samples

Activity concentrations of ^137^Cs in the litter samples, including those from the decontaminated areas, were more than several thousand becquerels per kilogram of dry weight in July 2015 (Table 1). The ^137^Cs activity concentrations decreased with time in both untreated and decontaminated areas. However, the ^137^Cs concentrations were still higher than several hundred becquerels per kilogram in both treatment areas in August 2017. Other than at the FR-2 site in July 2015, the difference in the ^137^Cs activity concentration in the litter samples between the two treatment areas was not statistically significant (using an unpaired *t*-test at the 5% significance level).

**Table 1:** Cesium-137 activity concentrations (Bq kg^−1^ dw) in litter and topsoil samples.

Sampling date	Sample	FR-1		FR-2	
		Untreated	Decontaminated	Untreated	Decontaminated
July 2015	Litter	5300 (3500)^a^	3200 (1200)^a^	4300 (800)^a^	2200 (400)^a^
	Litter F1	700 (500)	170 (80)	700 (400)	200 (40)^b^
	Litter F2	1200 (700)	600 (200)	800 (200)	630 (40)
	Litter F3	1300 (300)	1100 (400)	950 (10)	700 (300)
	Litter F4	7000 (4000)	5000 (2000)	5800 (1300)	3500 (400)
	Topsoil	1160 (70)	1000 (400)	690 (130)	500 (200)
September 2016	Litter	2200 (800)	1800 (900)	2000 (500)	1100 (900)
	Topsoil	1000 (500)	1000 (1100)	1900 (1100)	1000 (600)
August 2017	Litter	1180 (140)^c^	1400 (600)	700 (190)	1200 (400)
	Topsoil	430 (190)^c^	500 (300)	800 (600)	470 (50)

^a^Mean and standard deviation (in parentheses) of the three replicate samples (n = 3).

^b^Mean and standard deviation of two of the three replicate samples (n = 2), because the remaining sample showed ^137^Cs activity concentration lower than the detection limit (<210 Bq kg^−1^ dw).

^c^Only two replicate samples were available (n = 2).

Activity concentrations of ^137^Cs in the topsoil (0–5 cm) samples were lower than those of the litter samples (Table 1) and showed no significant difference (at a 5% significance level) between the untreated and decontaminated areas on any sampling occasion at either site.

### Inventory of ^137^Cs in litter and topsoil layers

**Figure 4:**
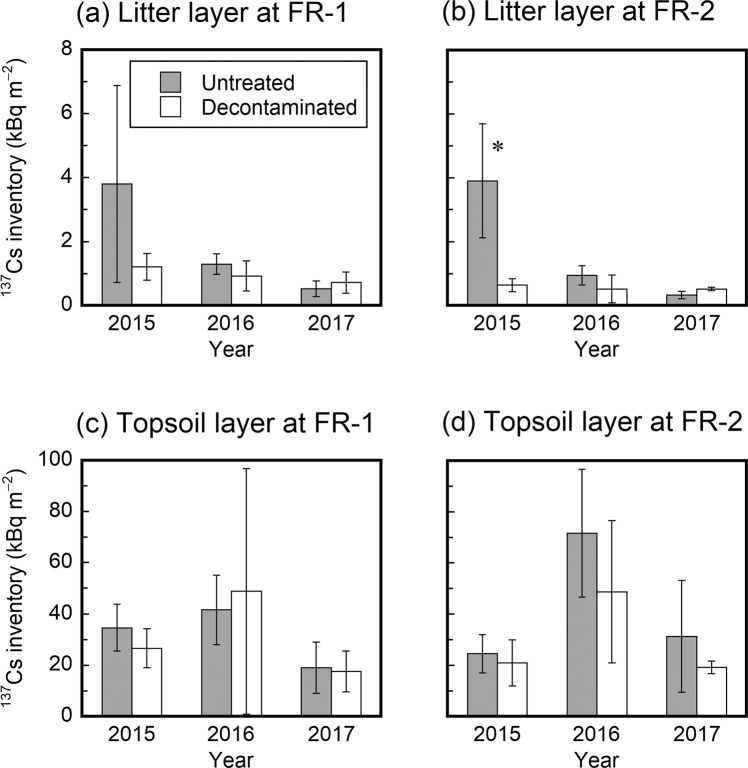
Temporal changes after decontamination (July 2014) in the ^137^Cs inventory in the litter (**a**,**b**) and topsoil (**c**,**d**) layers in the untreated and decontaminated areas at FR-1 and FR-2 sites. Asterisks indicate statistically significant differences.

The ^137^Cs inventory in the litter layer was estimated to be 0.3–3.9 kBq m^−2^ (Table S1 in Supplementary information) and decreased with time during the three-year observation period after decontamination, particularly in the untreated areas (Fig. 4). In July 2015, the ^137^Cs inventory in the litter layer was larger in the untreated areas than in the decontaminated areas at both sites, although the difference was significant (using an unpaired *t*-test at the 5% significance level) only for the FR-2 site. In subsequent years, the ^137^Cs inventory in the litter layer was similar between the two treatment areas at both sites.

The ^137^Cs inventory in the topsoil (0–5 cm) layer was estimated to be 18–72 kBq m^−2^ (Table S1), and showed no significant difference (at the 5% significance level) between the two treatment areas on each of the sampling occasions (Fig. 4). In contrast to the litter layer, no consistent trend of increasing or decreasing ^137^Cs inventory with time was observed for the topsoil layer.

These results indicate that most of the deposited ^137^Cs was in the topsoil layer on all sampling occasions at both sites, regardless of the treatment (untreated or decontaminated), while the litter layer retained only 1.3–14.9% of the total ^137^Cs inventory (see Table S1). Relatively high ^137^Cs retention in the litter layer (>10% of the total) was observed in the untreated areas on the first sampling occasion, July 2015 (Fig. 4). This could be attributed to the larger inventory of litter materials in the untreated areas compared with the decontaminated areas (Fig. 3). However, the difference in the retention ratio between the two treatment areas was not significant (at a 5% significance level) at either site on any sampling occasion. The difference in the total ^137^Cs inventory (litter and topsoil layers) between the two treatment areas was also not statistically significant at either site on any sampling occasion (Table S1).

### Distribution of ^137^Cs in litter fractions

**Figure 5:**
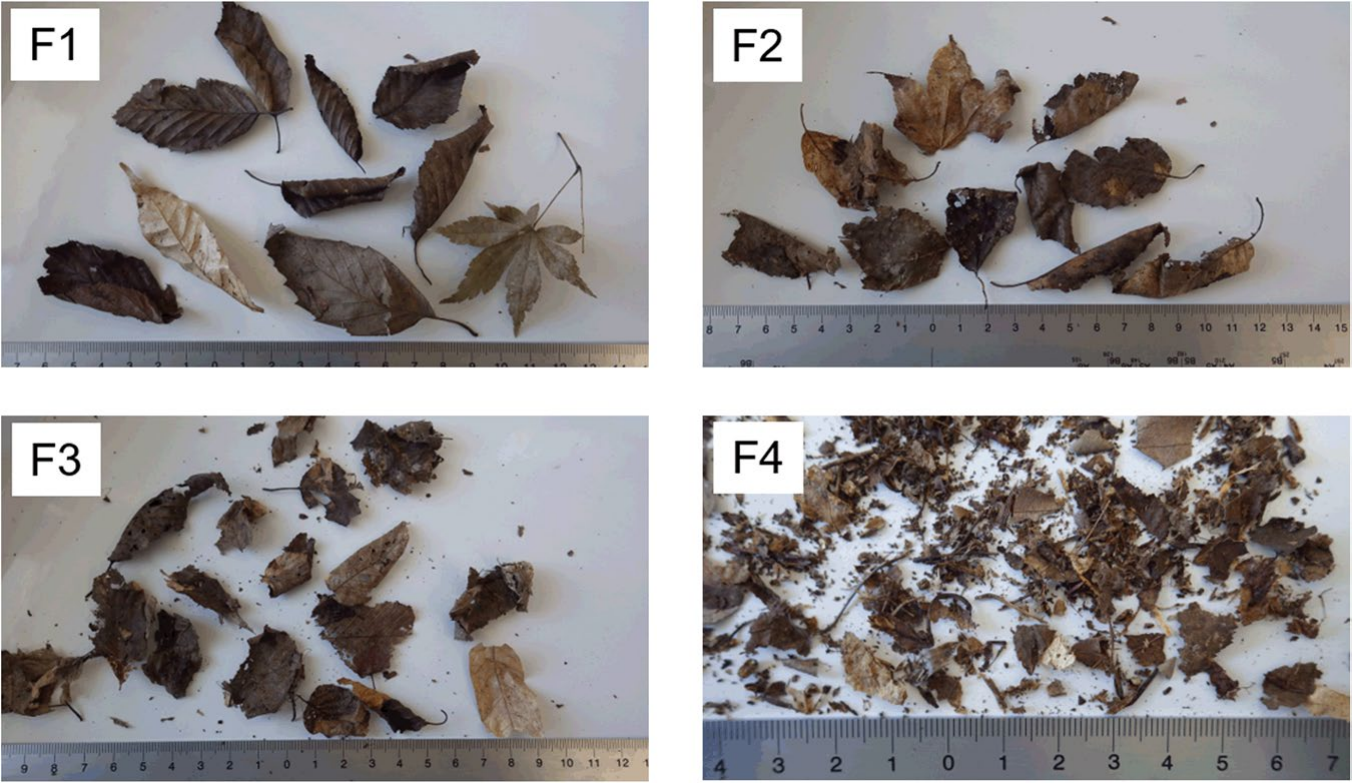
Examples of litter materials in the separated litter fractions. The fractions were obtained from the untreated area at FR-2 site and were differentiated by the degree of degradation. For more detailed definition, see the “Litter fractionation” section.

To investigate the distribution of ^137^Cs among different materials in the litter layer, the litter samples collected in July 2015 were separated into four fractions according to the physical status of the litter materials, using an index describing the degree of degradation (Fig. 5)^23^. The finely fragmented fraction, F4, was the largest, accounting for 53–69% of the total mass of litter materials (Table 2). In contrast, well-preserved leaf litter, F1, represented only a minor fraction of the total mass (<6%) in both untreated and decontaminated areas. Regardless of the treatment, ^137^Cs activity concentrations increased with decreasing size of litter materials (F1 to F4) and thus with increased degree of degradation^23^. As a result, the well-degraded F4 fraction was the most dominant in retaining ^137^Cs in the litter layer in both untreated and decontaminated areas (Table 2): more than 85% of the ^137^Cs in the litter layer was associated with the F4 fraction, whereas less than 1% was found in the well-preserved F1 fraction.

**Table 2:** Distributions of litter materials and litter-associated ^137^Cs among the four fractions (F1, F2, F3, and F4) of the litter layer in July 2015.

Site	Area	Mass of litter materials (%)				^137^Cs inventory (%)			
		F1^a^	F2	F3	F4	F1	F2	F3	F4
FR-1	Untreated	5.6 (1.3)^b^	22.2 (11.2)	7.2 (7.5)	65.1 (17.7)	0.7 (0.3)	5.7 (3.6)	3.0 (4.1)	90.5 (7.4)
	Decontaminated	2.1 (0.8)	18.0 (6.3)	27.2 (7.0)	52.8 (3.2)	0.2 (0.2)	3.7 (2.0)	10.4 (5.5)	85.7 (6.0)
FR-2	Untreated	1.9 (1.4)	13.4 (3.5)	15.9 (0.4)	68.8 (4.8)	0.3 (0.2)	3.0 (1.9)	3.7 (0.9)	93.1 (2.9)
	Decontaminated	3.5 (0.5)	21.1 (6.3)	22.1 (2.7)	53.3 (8.6)	0.2 (0.2)	6.4 (2.8)	7.8 (6.3)	85.6 (8.9)

^a^For an explanation of the litter fraction designations, see the “Litter fractionation” section.

^b^Mean and standard deviation (in parentheses) of the three replicate samples (n = 3).

### Activity concentrations of ^137^Cs in fresh leaf samples

**Figure 6:**
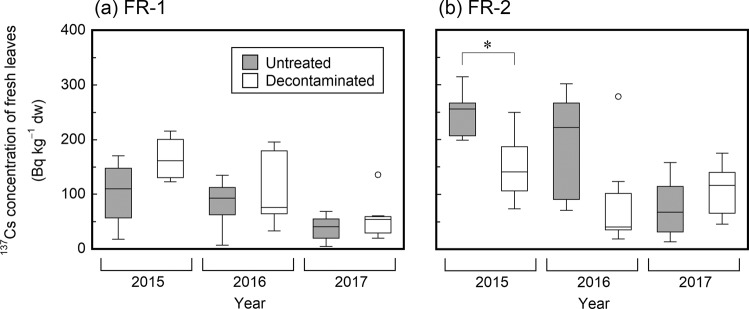
Temporal changes after decontamination (July 2014) in the ^137^Cs activity concentrations in tree leaves collected in the untreated and decontaminated areas at (**a**) FR-1 and (**b**) FR-2 sites. The trees were all broadleaved tree species, including *Carpinus*, *Acer amoenum*, *Ilex macropoda*, *Quercus serrata*, and *Styrax japonica*. Asterisks indicate statistically significant differences.

^137^Cs activity concentrations in fresh leaves of broadleaved tree species ranged from 5 to 315 Bq kg^−1^ dry weight (dw), and generally showed a decreasing trend with time (Fig. 6; data for each tree are available in Tables S2 and S3 in Supplementary information). There was no significant difference (at the 5% significance level) between the two treatment areas on any of the sampling occasions, other than a significantly higher ^137^Cs concentration in the untreated area than in the decontaminated area at the FR-2 site on the first sampling occasion (July 2015). No clear relationship was observed between the fresh leaf ^137^Cs concentration and tree species (Table S2 and S3). The ^137^Cs concentrations in fresh leaf samples were much lower compared with those of the litter and topsoil samples (see Table 1).

### Aggregated transfer factor (*T*_*ag*_) for ^137^Cs in tree leaves

Mean values of *T*_*ag*_ for ^137^Cs in tree leaves were in the order of 10^−3^ m^2^ kg^−1^ dw (Table 3), determined as the ratio of ^137^Cs activity concentration (in Bq kg^−1^ dw) in tree leaves divided by the total ^137^Cs inventory (in Bq m^−2^) in litter and topsoil layers^29,30^. In general, *T*_*ag*_ values were similar between the two treatment areas on each of the sampling occasions, although the values were considered significantly higher (at the 5% significance level) in the decontaminated area than in the untreated area at FR-1 in 2015 and at FR-2 in 2017. No consistent pattern of decreasing or increasing *T*_*ag*_ with time was observed during the three-year observation period.

**Table 3:** Aggregated transfer factor (*T*_*ag*_ in m^2^ kg^−1^ dw) for ^137^Cs in tree leaves.

Site	Date	Untreated area			Decontaminated area			P^b^
		Geometric mean	Range	N^a^	Geometric mean	Range	N	
FR-1	July 2015	2.0 × 10^−3^	4.7 × 10^−4^ –4.4 × 10^−3^	4	5.8 × 10^−3^	4.4 × 10^−3^ –7.7 × 10^−3^	4	<0.05
	September 2016	1.4 × 10^−3^	1.6 × 10^−4^ –3.1 × 10^−3^	7	1.9 × 10^−3^	6.6 × 10^−4^ –3.9 × 10^−3^	7	0.63
	August 2017	1.5 × 10^−3^	2.5 × 10^−4^ –3.5 × 10^−3^	8	2.5 × 10^−3^	1.1 × 10^−3^ –7.4 × 10^−3^	8	0.22
FR-2	July 2015	8.6 × 10^−3^	7.0 × 10^−3^ –1.1 × 10^−2^	5	6.4 × 10^−3^	3.4 × 10^−3^ –1.2 × 10^−2^	6	0.25
	September 2016	2.4 × 10^−3^	9.8 × 10^−4^ –4.2 × 10^−3^	6	1.2 × 10^−3^	3.8 × 10^−4^ –5.6 × 10^−3^	7	0.33
	August 2017	1.8 × 10^−3^	4.3 × 10^−4^ –5.0 × 10^−3^	8	5.0 × 10^−3^	2.3 × 10^−4^ –8.8 × 10^−3^	8	<0.05

^a^Number of samples.

^b^P-value for the unpaired *t*-test to compare the means of *T*_*ag*_ in the untreated and decontaminated areas.

## Discussion

The results of the present study clearly demonstrate that the litter-removal decontamination conducted approximately 3.3 years after the Fukushima NPP accident was ineffective in reducing the ^137^Cs contamination levels in a deciduous broadleaved forest in Japan. Litter removal decreased the inventories of litter materials and litter-associated ^137^Cs in the decontaminated areas one year after the decontamination, but the effects were not observed in the following years (Figs. 3 and 4). This could be explained by the rapid turnover of litter material in Japanese forest ecosystems. In the present study, even in the untreated areas, less than 15% of the deposited ^137^Cs was retained in the litter layer by July 2015 (Table S1), and most of the litter-associated ^137^Cs occurred in association with well-degraded litter material (Table 2). In June 2011, three months after the accident, the ^137^Cs inventories in the litter layers at the FR-1 and FR-2 sites were determined to be 44.1 and 25.8 kBq m^−2^, respectively^31^, which were approximately an order of magnitude greater than the ^137^Cs inventories in July 2015 (Fig. 4 and Table S1). It has recently been estimated that the ^137^Cs inventory in the litter layers of this forest decreases over time with an ecological half-life of approximately one year^32^. Taken together, these observations suggest that most of the deposited ^137^Cs had already migrated from the litter layer to the underlying mineral soil through litter decomposition by July 2014 when the litter-removal decontamination was conducted^32–36^. This is the most likely cause of the litter-removal decontamination employed here being ineffective in reducing the inventories of ^137^Cs in both the litter and topsoil layers of this forest. The negative result for decontamination is therefore largely due to the low ^137^Cs retention capability of the litter layer. This radioecological characteristic of Japanese forest ecosystems^32^ contrasts with European forest ecosystems affected by the Chernobyl NPP accident, where litter layers retained the largest proportion of deposited ^137^Cs for over a decade^11,15,16,37–39^. Negligible decontamination effects have also been reported by Ayabe *et al*.^27^, who conducted litter-removal decontamination in a mixed broadleaved and coniferous forest in May 2015, approximately 4.2 years after the Fukushima NPP accident.

Effects of litter-removal decontamination on vegetation were also not obvious. The ^137^Cs activity concentrations in fresh leaves were generally similar across the two treatments, although a lower ^137^Cs activity concentration was observed in the decontaminated area than in the untreated area at the FR-2 site in July 2015 (Fig. 6). The aggregated transfer factor (*T*_*ag*_) values for ^137^Cs in tree leaves were also generally similar across treatments (although estimated to be higher in the decontaminated area than in the untreated area in some cases) (Table 3). These results seem reasonable given that, prior to decontamination, a large proportion of the deposited ^137^Cs was immobilized in the surface mineral soil via its interactions with soil constituents^40–45^. This behavior could reduce ^137^Cs availability for root uptake by plants^15,46,47^ to a similar extent in both the untreated and decontaminated areas. Activity concentrations of ^137^Cs in fresh leaves can also be affected by the internal translocation of ^137^Cs from the tree body (the trunk, branches, and bark) to newly emerging leaves^48,49^. However, it is likely that such a process within a tree is not materially affected by the removal of litter.

The transfer of ^137^Cs from soil to tree can be influenced by many factors, such as the initial contamination process, the season, the tree’s physiology and age, and the ^137^Cs distribution and availability in the soil^15,20,50–52^. Therefore, *T*_*ag*_ values change over time as a result of changes in such factors, and normally decrease until ^137^Cs cycling within a forest ecosystem reaches a quasi-steady state^29,30^. The *T*_*ag*_ values obtained in the present study did not show any time-dependent trends during the three-year observation period (Table 3), indicating that ^137^Cs fluxes in this forest had almost stabilized through natural ecosystem processes by the first sampling occasion (approximately 4.3 years after the accident). Again, this suggests that the mobility and availability of ^137^Cs in this forest decreased rapidly owing to the rapid shift in the main reservoir of ^137^Cs from the litter layer to the underlying mineral soil, and that, as a result, ^137^Cs recycling within the forest (i.e., the uptake of ^137^Cs by trees from the litter layer and the redeposition of ^137^Cs onto the forest floor via annual leaf fall) was inhibited^43,53^. This is also substantiated by the observation of low ^137^Cs activity concentrations in newly emerged leaves (Fig. 6) and the low ^137^Cs inventories in the materials recently added to the litter layers (Table 2) in this forest. The geometric means of the *T*_*ag*_ values obtained in this study were 1.1 × 10^−3^ to 8.6 × 10^−3^ m^2^ kg^−1^ (Table 3). The values were comparable with those (2.5 × 10^−3^ to 1.2 × 10^−2^ m^2^ kg^−1^) measured under apparent steady state conditions in European deciduous broadleaved forests affected by the Chernobyl NPP accident^29,30^. In Fukushima, Nakai et al^54^. have reported higher *T*_*ag*_ values (6 × 10^−3^ to 6.2 × 10^−2^ m^2^ kg^−1^) for a range of deciduous species one and a half years after the Fukushima NPP accident.

This decontamination study was conducted in a deciduous broadleaved forest in Fukushima. It is worthwhile to ask whether litter-removal decontamination is also ineffective in reducing ^137^Cs contamination of evergreen coniferous forests in Fukushima. Deciduous broadleaved forests (49.1% of the forested area) and evergreen coniferous forests (47.7%) were the dominant forest types in the area heavily contaminated with ^137^Cs from the Fukushima NPP accident^6^. These forests greatly differ in their tree phenology and the initial contamination process (e.g., initial interception of ^137^Cs by the tree canopy and subsequent input to the forest floor via litterfall and throughfall^55^); it is therefore likely that the behavior of ^137^Cs in forest ecosystems after atmospheric fallout differs depending on the forest type^25,36,52^. An investigation conducted in an evergreen coniferous forest affected by the Fukushima NPP accident showed a slower migration of ^137^Cs from the litter layer to the mineral soil compared with deciduous broadleaved forest^25^. Taking into account the different ^137^Cs retention behavior in the litter layers, a limited but positive effect of litter-removal decontamination may be expected for evergreen coniferous forests in Japan. Sanderson *et al*.^26^ have reported that litter-removal decontamination conducted in September 2013 resulted in a 29% reduction of the ambient dose rate in a Japanese cedar plantation affected by the Fukushima NPP accident.

What is likely to be the most effective decontamination method for Japanese forest ecosystems? As forest decontamination operations are labor-consuming, expensive, and involve the risk of exposing the workers to radiation, they must be planned carefully^3^. Our study, however, suggests that in the case of Japanese forest ecosystems, litter-removal decontamination can only be successful if it is implemented quickly after forest contamination. The efficiency of litter-removal decontamination largely depends on the time between its implementation and the initial contamination, because of the low ^137^Cs retention capability of the litter layer^32^. According to a time series investigation of various types of forest in Fukushima^56^, it is estimated that 41–84% of the deposited ^137^Cs could have been removed from the forest soil environment if litter-removal decontamination had been carried out within four months of the accident. One should note, however, that the deposition of ^137^Cs on the forest floor is quite likely delayed by initial canopy interception of fallout, particularly in evergreen coniferous forests. Kato *et al*.^55^ have estimated that approximately 70% of the ^137^Cs fallout was initially intercepted by the tree canopy in Japanese cedar forests during the Fukushima NPP accident, and a significant fraction of the intercepted ^137^Cs was deposited onto the forest floor via throughfall and litterfall in the year following the accident. Notwithstanding this delay, a shorter time interval (<1 year for deciduous broadleaved forests and 1–2 years for evergreen coniferous forests) between the initial deposition and the implementation of litter-removal decontamination is required to achieve successful decontamination for Japanese forest ecosystems. This contrasts with the belief that litter-removal decontamination is effective for the first 2–5 years after the initial fallout^19,20,57^, a claim based on post-Chernobyl studies for European forest ecosystems.

Although the low ^137^Cs retention capability of litter layers in Japanese forest ecosystems results in the rapid reduction in ^137^Cs recycling within a forest without any artificial interventions, the surface layers of mineral soil act as a long-term reservoir of ^137^Cs^36,53,58^ and can thus not only be a prolonged source of external radiation exposure to the population^59,60^ but also be a secondary source of ^137^Cs contamination via redistribution of soil-associated ^137^Cs through physical processes^61,62^. Therefore, although the removal of the litter layer is no longer effective in reducing the ^137^Cs contamination in Japanese forest ecosystems nine years after the accident, the removal of the surface soil layer may still merit consideration as an effective decontamination method^7,21,53^. The negative consequences of this method must be carefully considered, however, including the disturbance of the ecological balance of the forest ecosystems (e.g., disruption of nutrient cycles), the risk of soil erosion in the decontaminated soil surfaces during decontamination and the subsequent vegetation recovery period particularly in mountainous and hilly regions with steep terrain, and the generation of large quantities of radioactive waste^10,19^.

## Methods

### Study sites

This study was conducted in a deciduous broadleaved forest (elevation: 250 m, area: 38.8 ha) located in the southwestern part of the city of Fukushima, approximately 70 km northwest of the Fukushima Daiichi NPP (Fig. 1). The forest is on flat terrain and has a 3.1 km promenade connected to a public park, offering recreational opportunities for many people, including hiking, birding, and camping. The forest consists of various broadleaf tree species such as *Carpinus*, *Acer amoenum*, and *Ilex macropoda*. The bulk of deciduous leaf fall (litterfall) occurs in autumn (October and November), hence the trees were leafless at the time of the Fukushima NPP accident (March 2011). The soil in this forested area has been classified as Fluvisol, using the classification of the Food and Agriculture Organization of the United Nations (FAO). The mean annual temperature and precipitation at the nearest meteorological observation station are 13.3 °C and 1234 mm, respectively. Snowfall is usually observed from December to March, and the mean annual snowfall is 113 cm.

The vertical distribution of ^137^Cs in the soil profile has been investigated at two sites (FR-1 and FR-2) within this forest since the Fukushima NPP accident, and the sites were found to have levels of ^137^Cs deposition ranging from 40 to 80 kBq m^−2 ^ ^25,31,56^. Since these levels are markedly higher than the global ^137^Cs fallout from atmospheric nuclear weapons testing (estimated at 2–4 kBq m^−2^)^58,63^, the ^137^Cs observed in the present study was considered to originate primarily from the Fukushima NPP accident. This has been confirmed by ^134^Cs/^137^Cs activity ratios of litter and soil samples collected at these sites since the accident^25,31,56^.

The FR-1 site is situated in the middle of the forest whereas the FR-2 site is in the north of the forest; the distance between the two sites is approximately 250 m. For more detail on the site characteristics, including the physicochemical properties of the soils, see Koarashi *et al*.^31^. In this forest, management practices such as grass cutting have long been kept to a minimum to protect rare wild grasses and flowers. However, in the decontamination operation conducted in July 2014 (three years and four months after the Fukushima NPP accident) understory grasses were cut and removed from almost the entire area of the forest (see next section for details).

### Plot establishment and decontamination operation

At each of the two sites, a plot (untreated plot) was established prior to the decontamination operation in July 2014 (Fig. 2a). The plots were approximately 20 × 20 m and 15 × 15 m for the FR-1 and FR-2 sites, respectively, and were not subjected to any disturbance during the decontamination operation. The plots have been kept intact and therefore can be defined as “untreated areas” in the present study (Fig. 2b). The entire area of the forest other than the two plots (and some small areas containing rare wild grasses and flowers) was decontaminated in July 2014 by removing the forest floor litter layer with rakes and brooms and can thus be defined as “decontaminated area.” Understory grasses in the decontaminated area were also cut and removed during the decontamination operation.

### Sample collection and treatment

Following the decontamination in July 2014, samples were collected on three occasions (July 2015, September 2016, and August 2017) in the untreated and the adjacent decontaminated areas of the two sites (FR-1 and FR-2) (Fig. 2a). Sample collection was conducted prior to the litterfall events (October and November) on each occasion. Three replicate litter samples were collected manually from an area of 900 cm^2^ (30 × 30 cm), each being randomly selected within each of the untreated and decontaminated areas. Three replicate topsoil (0–5 cm) samples were then collected using a cylindrical soil sampler (5 cm in diameter and 5 cm in depth) from the soil surface where the litter layer was collected^25^. The depth range (0–5 cm) of the soil sampling was chosen on the basis of studies showing that most of the total ^137^Cs inventory is located within the litter and the upper 5 cm of soil for several years after deposition^31,53^. Fresh leaf samples were collected from standing trees in the untreated and decontaminated areas, using a pair of pole-type pruning shears. The trees were all broadleaved tree species, including *Carpinus*, *Acer amoenum*, *Ilex macropoda*, *Quercus serrata*, and *Styrax japonica*.

The collected samples were transported to the laboratory and dried to a constant weight at room temperature. After removing coarse woody debris (fallen branches and twigs), the litter samples were finely chopped using a mixer to obtain a homogeneous particle size. The litter samples collected in July 2015 were subjected to litter fractionation before conducting this treatment (see next section for details of litter fractionation). The soil samples were sieved through a 2-mm mesh, and both fractions (<2 mm and >2 mm) were weighed to determine the bulk density and gravel content of the soil. The fresh leaf samples were finely chopped as described above for the litter.

### Litter fractionation

To investigate the distribution of ^137^Cs among the different types of materials in the litter layers, the litter samples collected in July 2015 were separated into four fractions (Fig. 5) according to the physical status of the litter materials, using an index describing the degree of degradation^23,32^. Fraction F1 consisted of leaves showing no visible signs of degradation. Fractions F2 and F3 consisted of leaves that were more or less chipped or degraded; these two fractions were differentiated by leaf size:>3 × 3 cm for the F2 fraction and <3 × 3 cm for the F3 fraction. Fraction F4 consisted mainly of fine leaf fragments (<1 × 1 cm in size), including petioles detached from leaves and macroscopically unrecognizable materials. The degree of degradation of litter materials is closely related to their size and increases in the order of F1 < F2 < F3 < F4 in the fractionation method^23^.

### Radiocesium analysis

The activity concentrations of ^137^Cs in the litter, soil, and fresh leaf samples were determined using gamma ray spectrometry, and their values were expressed in activity per unit dry weight (Bq kg^−1^ dw). Samples were sealed in plastic tubes (5 cm diameter, 7 cm height) and analyzed for ^137^Cs using a high-purity coaxial germanium detector (model GEM25P4–70, ORTEC, USA) at the Japan Atomic Energy Agency. The detector was calibrated with standard gamma sources (each with a relative uncertainty of ~5% for ^137^Cs) with different sample heights. The measurement times ranged from 3,600 to 690,000 s, depending on the sample type and the radiocesium concentration of the sample; this allowed us to obtain ^137^Cs concentration values with relative errors of <5% in most cases, with some exceptions for samples having low ^137^Cs concentrations (in particular, fresh leaf samples). The ^137^Cs activity concentrations were corrected for radioactive decay to the sampling date.

The ^137^Cs inventory (Bq m^−2^) in the litter (*I*_*L*_) and topsoil (*I*_*S*_) layers were estimated as

1 $${I}_{L}={A}_{L}\cdot {M}_{L},$$

2 $${I}_{S}={A}_{S}\cdot B\cdot (1-g)\cdot d,$$

where *A*_*L*_ is the ^137^Cs activity concentration (Bq kg^−1^ dw) of the litter sample, *M*_*L*_ is the amount of litter material per unit area (kg m^−2^) in the litter layer, *A*_*S*_ is the ^137^Cs activity concentration (Bq kg^−1^ dw) of the soil (<2 mm) sample, *B* is the bulk density of the soil (kg m^−3^), *g* is the gravel (>2 mm) content of the bulk soil (kg kg^−1^), and *d* is the thickness (m) of the topsoil layer (i.e., 0.05 m)^25^.

### Determining aggregated transfer factors for ^137^Cs in tree leaves

Aggregated transfer factors (*T*_*ag*_ in m^2^ kg^−1^ dw) are frequently used to quantify ^137^Cs availability to various types of natural vegetation in forest ecosystems^29,30^. *T*_*ag*_ is defined as the ratio of ^137^Cs activity concentration in a tree component (in Bq kg^−1^ dw) divided by the total ^137^Cs deposition in the soil (in Bq m^−2^). The concept of *T*_*ag*_ provides a reasonable empirical measure to normalize ^137^Cs accumulation in the tree component, regardless of site-specific variations in the ^137^Cs vertical distribution and availability in the soil profile^29^. In the present study, *T*_*ag*_ values were estimated for the untreated and decontaminated areas on each of the sampling occasions, by dividing the observed ^137^Cs activity concentrations in tree leaf samples by the ^137^Cs inventory in the soil (litter and topsoil layers).

## Supplementary information

Supplementary Information.
