# Supplementary material for: Effectiveness of decontamination by litter removal in Japanese forest ecosystems affected by the Fukushima nuclear accident
Source: Sci Rep. 2020 Apr 20;10:6614. doi: 10.1038/s41598-020-63520-8 (PMC7171154; doi:10.1038/s41598-020-63520-8)
Supplement: Supplementary file 1 — Supplementary Information. [file 41598_2020_63520_MOESM1_ESM.pdf]

Supplementary Information

Title: Effectiveness of decontamination by litter removal in Japanese forest ecosystems affected by the Fukushima nuclear accident

Authors: Jun Koarashi, Mariko Atarashi-Andoh, Syusaku Nishimura & Kotomi Muto

Table S1

Cesium-137 inventory in the litter and topsoil (0–5 cm) layers in the untreated and decontaminated areas at FR-1 and FR-2 sites

Site	Sampling date	Area (treatment)	^{137}Cs inventory (kBq m^{-2})			Litter/Total (%)
			Litter layer	Topsoil	Total	
FR-1	July 2015	Untreated	3.8 (3.1)[a]	35 (9)[a]	39 (9)[a]	10.2 (7.2)[a]
		Decontaminated	1.2 (0.4)	27 (8)	28 (7)	4.7 (2.5)
	September 2016	Untreated	1.3 (0.3)	42 (14)	43 (13)	3.4 (1.6)
		Decontaminated	0.9 (0.5)	49 (48)	50 (48)	2.7 (1.9)
	August 2017	Untreated	0.5 (0.2)[b]	19 (10)[b]	20 (10)[b]	3.4 (2.9)[b]
		Decontaminated	0.7 (0.3)	18 (8)	18 (8)	4.0 (1.1)
FR-2	July 2015	Untreated	3.9 (1.8)	25 (7)	28 (6)	14.9 (8.9)
		Decontaminated	0.6 (0.2)	21 (9)	22 (9)	3.7 (2.7)
	September 2016	Untreated	1.0 (0.3)	72 (25)	73 (25)	1.4 (0.6)
		Decontaminated	0.5 (0.4)	49 (28)	49 (28)	1.4 (1.2)
	August 2017	Untreated	0.3 (0.1)	31 (22)	32 (22)	1.3 (0.8)
		Decontaminated	0.5 (0.0)	19 (3)	20 (2)	2.6 (0.5)

[a]Mean and standard deviation (in parentheses) of the three replicate samples (N =3).

[b]Only two replicate samples were available (N = 2).

Table S2

Cesium-137 activity concentrations of fresh leaves of broadleaved tree species at FR-1 site

Sampling date	Untreated area		Decontaminated area	
	Species	^{137}Cs conc. (Bq kg^{-1} dw)	Species	^{137}Cs conc. (Bq kg^{-1} dw)
July 2015	*Carpinus*	96 ± 8[a]	*Ilex macropoda*	186 ± 16[a]
	Styrax japonica	18 ± 3	*Carpinus*	123 ± 5
	Acer pictum	124 ± 6	*Acer amoenum*	216 ± 10
	Acer palmatum	171 ± 6	*Acer amoenum*	138 ± 8
	**Average**	**102 (64)**[b]	**Average**	**166 (43)**[b]
September 2016	*Carpinus*	93 ± 1	*Carpinus*	66 ± 4
	Carpinus	35 ± 5	*Ilex macropoda*	33 ± 3
	Carpinus	90 ± 3	*Ilex macropoda*	63 ± 2
	Carpinus	135 ± 2	*Styrax japonica*	196 ± 10
	Fagus crenata	7 ± 1	*Acer amoenum*	190 ± 4
	Acer amoenum	128 ± 2	*Acer amoenum*	76 ± 2
	Acer pictum	97 ± 1	*Acer amoenum*	170 ± 2
	**Average**	**83 (47)**	**Average**	**113 (69)**
August 2017	*Carpinus*	58 ± 1	*Carpinus*	20 ± 1
	Carpinus	22 ± 1	*Carpinus*	61 ± 3
	Carpinus	51 ± 2	*Acer*	58 ± 2
	Acer	31 ± 1	*Acer amoenum*	136 ± 3
	Acer	52 ± 1	*Ilex macropoda*	50 ± 3
	Acer amoenum	69 ± 1	*Ilex macropoda*	35 ± 2

Styrax japonica	18 ± 3	*Ilex macropoda*	24 ± 2
Styrax japonica	5 ± 1	*Ilex macropoda*	58 ± 2
**Average**	**38 (23)**	**Average**	**55 (36)**

[a]Errors represent counting errors in the radiation measurement.

[b]Mean and standard deviation (in parentheses) of the samples (N = 4–8).

Table S3

Cesium-137 activity concentrations of fresh leaves of broadleaved tree species at FR-2 site

Sampling date	Untreated area		Decontaminated area	
	Species	^{137}Cs conc. (Bq kg^{-1} dw)	Species	^{137}Cs conc. (Bq kg^{-1} dw)
July 2015	*Ilex macropoda*	207 ± 9[a]	*Carpinus*	138 ± 11[a]
	Quercuc serrata	256 ± 5	*Styrax japonica*	74 ± 12
	Carpinus	199 ± 6	*Quercuc serrata*	107 ± 4
	Acer amoenum	267 ± 11	*Ilex macropoda*	145 ± 10
	Acer amoenum	315 ± 10	*Acer amoenum*	187 ± 16
			Acer amoenum	250 ± 11
	**Average**	**249 (47)[b]**	**Average**	**150 (62)[b]**
September 2016	*Carpinus*	71 ± 1	*Carpinus*	41 ± 2
	Carpinus	213 ± 3	*Carpinus*	80 ± 2
	Ilex macropoda	91 ± 6	*Carpinus*	37 ± 3
	Quercuc serrata	302 ± 2	*Ilex macropoda*	19 ± 2
	Acer amoenum	267 ± 4	*Quercus crispula*	34 ± 1
	Acer amoenum	232 ± 5	*Acer amoenum*	124 ± 3
			Acer amoenum	279 ± 5
	**Average**	**196 (94)**	**Average**	**88 (91)**
August 2017	*Carpinus*	90 ± 2	*Carpinus*	50 ± 2
	Acer amoenum	47 ± 1	*Acer amoenum*	150 ± 6
	Acer amoenum	140 ± 2	*Acer amoenum*	131 ± 4
	Ilex macropoda	89 ± 3	*Acer amoenum*	121 ± 2

Ilex macropoda	31 ± 2	*Ilex macropoda*	82 ± 1
Ilex macropoda	33 ± 1	*Ilex macropoda*	46 ± 1
Quercuc serrata	158 ± 2	*Ilex macropoda*	113 ± 1
Shirakia japonica	14 ± 1	*Quercuc serrata*	175 ± 2
**Average**	**75 (53)**	**Average**	**108 (46)**

[a]Errors represent counting errors in the radiation measurement.

[b]Mean and standard deviation (in parentheses) of the samples (N = 5–8).
